# Multisystemic Beryllium Disease: An Exceptional Case Revealed by a Urinary Tract Granulomatosis

**DOI:** 10.3390/ijms25158166

**Published:** 2024-07-26

**Authors:** Lucas Jacobs, Maxime Taghavi, Jennifer Fallas, Caroline Geers, Mark Libertalis, Julie Smet, Joëlle Nortier, Maria do Carmo Filomena Mesquita

**Affiliations:** 1 Department of Internal Medicine, Brugmann University Hospital, Université libre de Bruxelles, 1020 Bruxelles, Belgium; 2 Department of Nephrology and Dialysis, Brugmann University Hospital, Université libre de Bruxelles, 1020 Bruxelles, Belgium; 3 Department of Pathology, Institut Jules Bordet, Université libre de Bruxelles, 1070 Bruxelles, Belgium; 4 Department of Pathology, Brugmann University Hospital, Université libre de Bruxelles, 1020 Bruxelles, Belgium; 5 Department of Nephrology and Dialysis, Etterbeek-Ixelles Hospital, 1050 Brussels, Belgium; 6 Immunology Department, LHUB-ULB, Université libre de Bruxelles, 1000 Brussels, Belgium

**Keywords:** berylliosis, beryllium, granuloma, sarcoidosis, tubulointerstitial nephritis

## Abstract

Chronic beryllium disease (CBD), or berylliosis, is an interstitial lung disease caused by the chronic inhalation of finely particulate beryllium, frequently mistaken for sarcoidosis. It is rarely associated with skin nodular lesions, asymptomatic granulomatous hepatitis or calcium nephrolithiasis. To date, it has never been reported as a diffused multi-organ granulomatous disease. A 60-year-old Pakistani man, a former excavation worker with ancient history of suspected sarcoidosis, underwent a left nephroureterectomy for suspected papillary kidney carcinoma. The histopathological analysis showed a benign non-necrotic granulomatous infiltration of the renal pelvis and ureter. Six months later, he suffered from two consecutive episodes of acute kidney failure. Bladder biopsies found similar noncaseous granulomatosis and kidney biopsies showed interstitial nephritis. Known for suspected asthma, sleep apnea, and usual interstitial pneumonia, the patient would regularly consult for episodes of pyrexia, chills, nocturnal coughing, and wheezing. As kidney function gradually worsened, he ultimately started hemodialysis and was transferred to our facility. A positive blood beryllium lymphocyte proliferation test confirmed the diagnosis of CBD. This original report is the first description of multi-organ berylliosis with diffused urothelial granulomatosis and pseudo-tumor. The patient’s pulmonary disease is minimal compared with renal and urinary tract involvement, eventually responsible for end-stage kidney disease. Berylliosis usually responds to glucocorticoids. This case report highlights the importance of evoking the diagnosis of CBD in the presence of any granulomatosis, even extra-thoracic, especially if associated with pulmonary symptoms, however atypical.

## 1. Introduction

First described in 1946, chronic beryllium disease (CBD), so-called ‘berylliosis’, is a disease known to affect the lungs following the chronic inhalation of fine soluble particles of beryllium (Be) and its salts. Often misdiagnosed for sarcoidosis, berylliosis causes a chronic pulmonary granulomatous interstitial disease [1]. Occupational exposure to this metal occurs in a variety of industrial processes, affecting alloy workers, ceramic workers, and others [2,3]. The direct implantation of Be may also give rise to skin ulcers and nodules. Rarely, CBD has been associated with granulomatous hepatitis [4] and anecdotally affects kidneys.

Herein, we describe a unique case of berylliosis with multiple extra-thoracic organ involvements, among which ureteral granulomatous pseudo-tumors, diffused urothelial granulomatous infiltration, and renal interstitial nephritis, eventually lead to end-stage kidney failure and the need for chronic extra-renal epuration. We also provide a comprehensive review of beryllium and its human absorption, distribution and excretion, as well as further elaborating on berylliosis’ pathogenesis, sensitization, clinical and biological features, diagnostic tools, and treatment.

## 2. Case Presentation

We present the case of a 60-year-old Pakistani man, a former excavation and pipeline worker who lived in Saudi Arabia for 20 years. In recent years, he had resided in Greece, where he was diagnosed with pleural effusion and treated for six months for suspected pleural tuberculosis. During his time in Greece, he was also diagnosed with pulmonary sarcoidosis, but was not treated for it. Unfortunately, the initial work-up for his pulmonary disease remains unknown to this day. The patient had no history of smoking or alcohol consumption, and pantoprazole was his only medication.

**Figure 1:**
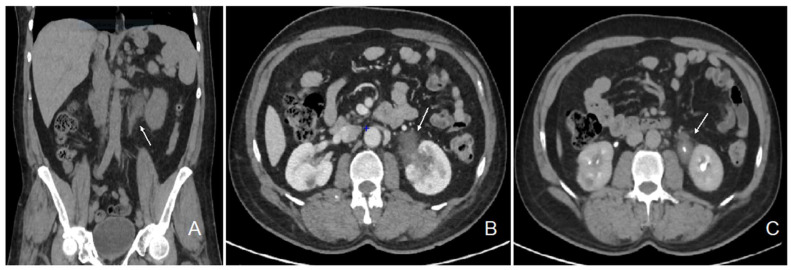
Abdominal CT scan images showing the pseudotumor of the left renal pelvis. Legend: coronal section, non-contrast phase (**A**); transversal section, nephrographic phase (**B**); transversal section, excretory phase (**C**); left ureteral tumor (white arrow).

The patient initially consulted our emergency service for recurrent left para-lumbar region pain. Abdominal computed tomography (CT) scan and renal sonography highlighted the presence of a polypoid tumor of the left ureter and renal pelvis causing moderate left uretero-hydronephrosis with moderate kidney failure (Figure 1) and locoregional enlarged lymph nodes. At the time, the right urinary tract was intact, and his biology showed a plasma creatinine of 1.35 mg/dL with an estimated glomerular filtration rate (eGFR) of 53 mL/min/1.73 m^2^ according to the CKD-EPI formula. Usual biological tests showed a normal calcium level and a mild 25 (OH) vitamin D deficiency with mild secondary hyperparathyroidism, and slight lymphopenia (Table 1). Urine cytology showed discrete to moderate nucleus abnormalities compatible with a low-grade papillary kidney carcinoma. Subsequently, the patient underwent a left nephroureterectomy.

**Figure 2:**
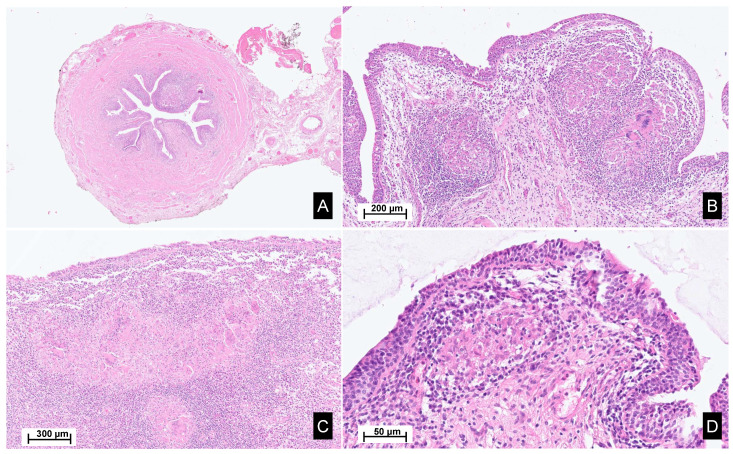
Histopathological images of the surgical material. Legend: Hematoxylin and eosin staining, sections from the ureter (**A**,**B**), renal pelvis and calyxes (**C**), and bladder (**D**), reveal poorly demarcated, non-necrotizing epithelioid granulomas within the lamina propria, accompanied by Langhans-type giant cells. These granulomas are associated with a mixed inflammatory infiltrate, rich in lymphocytes, plasma cells and eosinophils. Focally, a scarring fibrous reaction is observed.

A macroscopic pathological examination revealed a granulomatous aspect of the distal margin of the left ureter progressing towards the renal pelvis, holding a papillary lesion measuring 5.5 × 4.5 × 4 cm. Microscopic analysis revealed an intense inflammatory reaction of the proximal ureter and pelvic cavities. The inflammatory reaction coalesced into epithelioid granulomas with multinucleated giant cells. The epithelial lining showed a reactive aspect, with the formation of intraluminal pseudopolypoid formations without evidence of neoplastic tissue (Figure 2).

**Figure 3:**
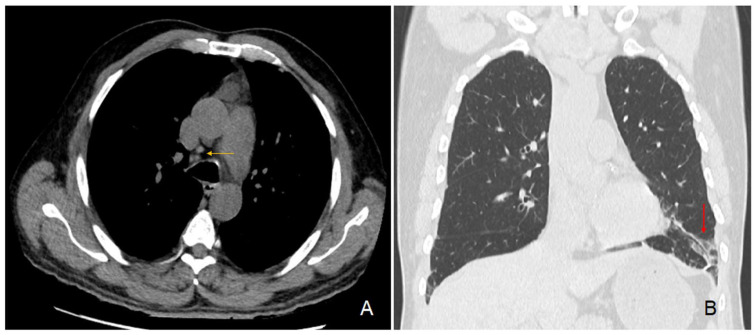
Chest CT scan images. Legend: transversal section (**A**) showing hilar lymphadenopathies (yellow arrow); coronal section (**B**) showing fibrous reticulations with bronchiolectasis of the pulmonary base (red arrow).

In parallel, the preoperative chest CT scan showed incidental signs of slight usual interstitial pneumonia characterized by fibrous reticulations and bronchiolectasis located at the pulmonary bases and small mediastinal lymphadenopathies (Figure 3). Since his surgery, the patient regularly consulted for recurrent pyrexia and chills, nocturnal coughing and wheezing, epistaxis, along with a 7 kg weight loss over 6 months. Subsequently diagnosed with asthma and sleep apnea syndrome, he was treated with inhaled fluticasone-vilanterol 92/22 μg and a continuous positive airway pressure device. A brain CT scan showed signs of chronic pansinusitis. Furthermore, our patient regularly consulted for recurrent episodes of pruriginous eczematiform rashes. Skin biopsies remained unspecific.

Within 6 months, our patient developed acute kidney injury (peak of plasma creatinine: 9.9 mg/dL; eGFR 6 mL/min). A double J stent was placed, partially resuming kidney function at discharge (nadir of plasma creatinine: 2.5 mg/dL; eGFR 25 mL/min).

**Figure 4:**
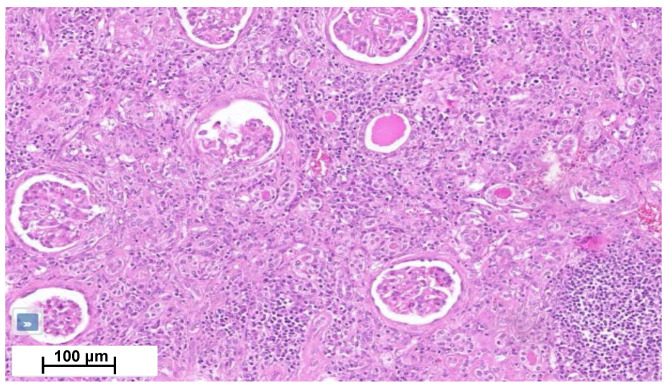
Kidney cortical section shows moderate chronic interstitial infiltrate rich in lymphocytes, with numerous sclerotic glomeruli and interstitial fibrosis.

Four months later, a second episode of acute kidney injury occurred (plasma creatinine reaching 7.8 mg/dL; eGFR 7 mL/min/1.73 m^2^). His urine samples showed a fluctuating albuminuria (0.5 to 1 g/g of urine creatinine), leukocyturia without cast formation, and microscopic hematuria without acanthocytes. In the absence of a clear diagnosis, the nephrology team performed a biopsy of his solitary right kidney. Renal biopsy showed an interstitial nephritis with lymphocyte infiltration without glomerular change (Figure 4). Immunofluorescence was unremarkable. Electron microscopy was not performed. Ziehl and Grocott staining were negative. Infectious work-up failed to detect tuberculosis (normal interferon-gamma release assays (IGRAs) and Mantoux tuberculin skin test for *Mycobacterium tuberculosis* (MBT), MBT PCR, and 3-week MBT cultures of urine samples). Also, ELISA assays for *Brucella* spp., *Bartonella henselae*, *Schistosoma mansoni*, *human immunodeficiency virus* and *Burkholderia pseudomallei* were negative. Antineutrophil cytoplasmic antibodies, antinuclear antibodies, angiotensin-converting enzyme and protein electrophoresis were normal, as well as IgG4 level (Table 1). Ophthalmological work-up was normal.

Bladder biopsies were later obtained and showed a fibrous tissue with numerous non-necrotic granulomas and multinucleated giant cells (Figure 2D). Special PAS and Zhiel staining did not reveal any pathogens. No coating nor malignancy was observed.

**Figure 5:**
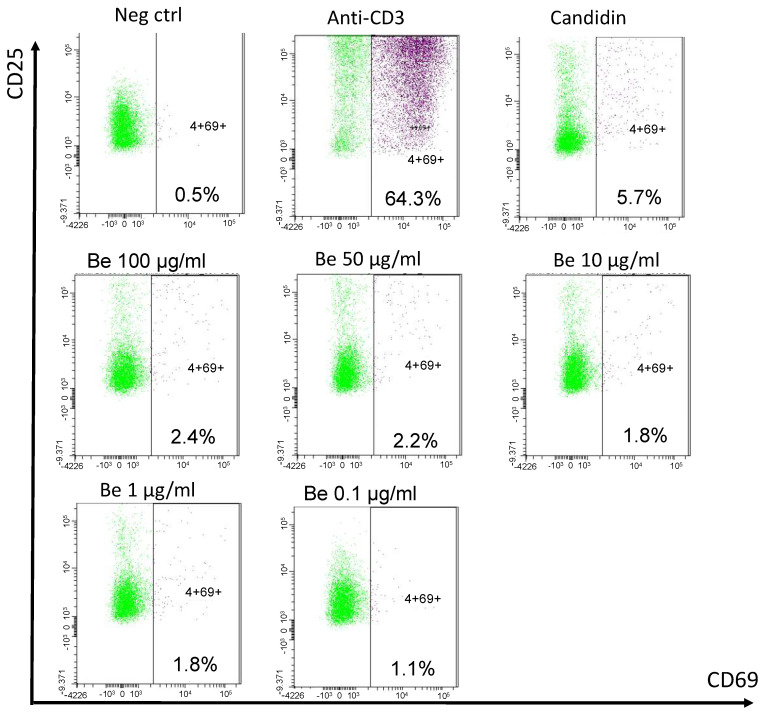
Blood beryllium lymphocyte proliferation test. Legend: PBMCs are incubated with negative (cell culture medium) and positive controls (anti-CD3 and Candidin), and with decreasing beryllium concentrations for 72 h. Activated CD69+ CD25+ CD4+ T cells are detected by cytometry after stimulation and stimulation index are calculated (% activated CD4+ T cells after stimulation/% activated CD4+ T cells in negative control).

Clinicians finally opted to treat the patient with a 6-month anti-tuberculous therapy. Corticosteroids were deemed risky in the event of a possible yet unproven tuberculosis. As kidney function parameters deteriorated, the patient started chronic hemodialysis. A year later, after having moved to another dialysis center, a blood beryllium lymphocyte proliferation test (BeLPT) was performed at 0.01, 0.1, 1, 10, 50, and 100 µg/mL of Be-sulfate tetrahydrate and was found positive with the four highest tested concentrations (Figure 5).

## 3. Discussion

### 3.1. Granulomatosis

A granuloma is a focal, compact collection of inflammatory cells, mostly mononuclear cells usually formed as a result of the persistence of a non-degradable product and hypersensitivity [1]. Granulomatous disorders encompass a large family of diseases comprising infections, vasculitis, immunological disorders, leucocyte oxidase defect, hypersensitivity, chemicals, drug reactions, and neoplasia [1,5]. Some metals and chemicals are known to induce the formation of granuloma, notably beryllium, zirconium, silica, aluminum, titanium, and talc [6,7]. Clinical findings, histological and biological analyses, as well as patients’ occupational history are therefore key to achieving adequate diagnosis and management.

### 3.2. Exposure to Beryllium and Toxicokinetics

The numerous properties of Be make it one of the most widely used metals in various fields such as aerospace, electronics, and electricity [2,3]. In medicine, some dental alloys may contain up to 2% Be [8]. Be is also used in the manufacturing of various sports items, such as hockey sticks, golf clubs, and bicycle hoops. Emeralds are composed of Be [9]. Therefore, there are occupations at risk of exposure to Be dust and vapors, beyond metal extraction mines. A study from 2006 identified 34 cases of berylliosis, in a cohort of 84 patients previously diagnosed as sarcoidosis with proven exposure to Be. Thirteen patients had been exposed to Be through dentistry, while other occupations included automotive work, aerospace, jewelry making, and others [2]. Thus far, Belgium has not experienced notable problems related to the toxicity of Be. Although this metal was used in the past in the fluorescent lamp industry, it was quickly abandoned after the description of its toxic effects on health. In 2021, only 3 people were identified and compensated for having Be-related diseases [10]. Worldwide, however, Be exposure is probably underestimated [11]; it is estimated that there are 67,000 workers exposed to beryllium in Europe [12].

After lung absorption of Be, short-term accumulation happens in the liver, especially when concentrations are high. In the long term, Be distributes to the lymph nodes and bones [13]. The excretion of absorbed Be is generally via urine [13] and intranuclear beryllium-rich structures can be detected within the renal convoluted tubular cells of rodents intoxicated with Be [14]. Urinary Be levels are not correlated with the intensity of exposure or the presence of berylliosis due to large individual variability and fluctuation in urinary excretion over time, influenced by stress, pregnancy, or infection [9,15].

### 3.3. Pathogenesis

CBD was initially considered as an immune-mediated disease [16]. However, the observation of cutaneous granulomas following intradermal injection of Be demonstrated the existence of delayed hypersensitivity [17]. Numerous animal models of Be exposure have been used to study CBD, although none fully incorporate all the features of the human form [18]. Knowledge about pathophysiology mostly comes from in vitro studies. The latter is complex, multifactorial, and likely still poorly understood.

**Figure 6:**
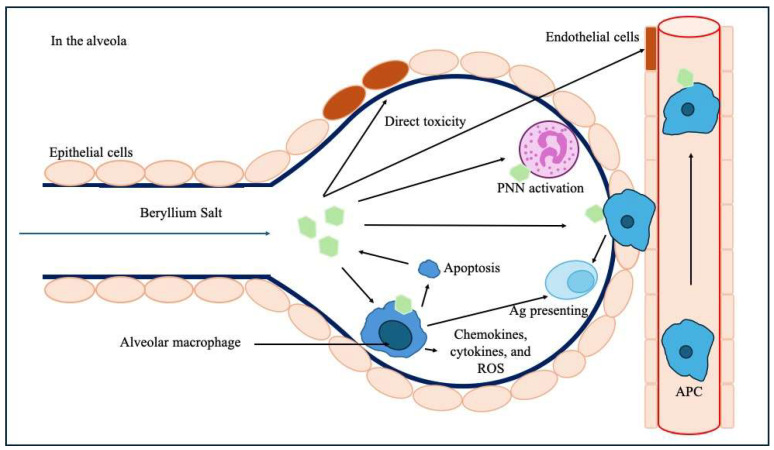
Pathophysiological mechanisms of Be-induced toxicity upon the alveolar–capillary barrier and subsequent immunological processes. Legend: Ag: antigen, APC: antigen-presenting cells, PNN: polynuclear neutrophil, ROS: reactive oxidative species.

It involves a direct toxic effect on lung epithelial cells and endothelial cells within the alveolo-capillary wall and decreases the ability of endothelial cells to prevent fibrosis [18]. Be could stimulate the migration of monocytes and blood lymphocytes to the lung and act as an adjuvant leading to accelerate the maturation of monocytes into a mature macrophage and antigen-presenting phenotype [19]. Macrophages lack the ability to metabolize Be, which ultimately leads to their apoptosis and the release of Be back into the microenvironment [20]. Macrophages can also act as antigen-presenting cells in the alveola, and Be induces the release of inflammatory chemokines, cytokines, and reactive oxidative species (ROS) from macrophages (Figure 6) [21].

**Figure 7:**
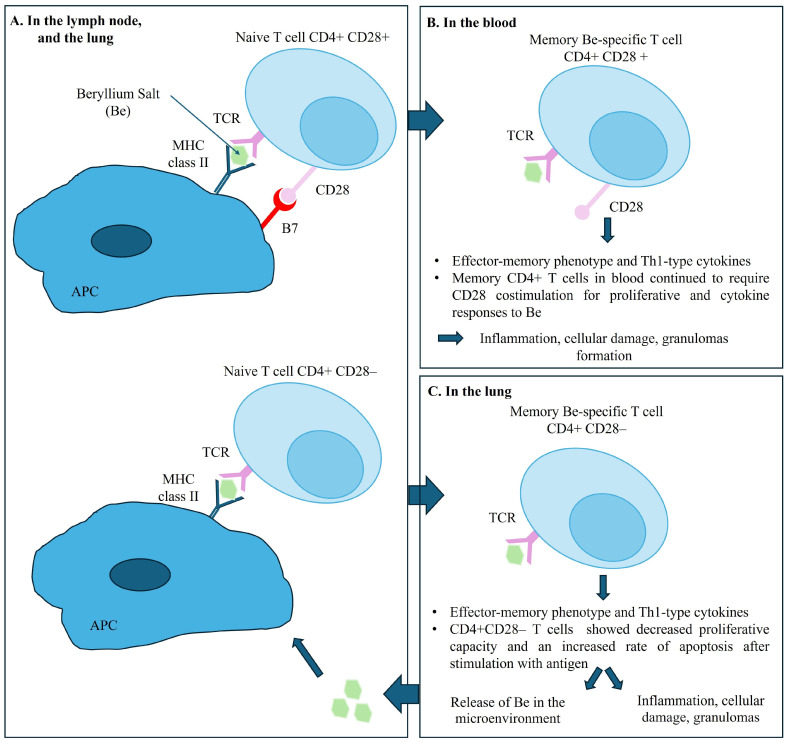
Pathogenesis of chronic beryllium disease. Legend: Panel (**A**) shows the immune response in the lymph nodes and the lungs, illustrating the interaction between naive T cells and APC upon engagement of T cell receptors (TCR) expressed on T cells with their cognate MHC II–peptide complex expressed on antigen-presenting cells (APC), with and without CD28 costimulation. Panel (**B**) shows the Memory CD4+ T cells in blood requiring CD28 costimulation for proliferative and cytokine responses to Be salt. Panel (**C**) shows the immune response in the lungs. Beryllium-specific CD4+ CD28- T cell expressing an effector-memory phenotype and the releasing of Th1-type cytokines when stimulated with Be salt.

Evidence indicates that the accumulation and activation of Be-specific CD4+ T cells in the lungs and in lymph nodes, is central to the pathogenesis of disease. The recognition of Be on the surface of APC and macrophages, upon engagement of T cell receptors (TCR) and class II MHC peptide complex triggers the differentiation of memory CD4+ T cells into effector CD4+ T cells [22] and cytokine secretion. Be-specific CD4+ T cells exhibit a cytokine profile oriented towards a Th1-type immune response involving interferon gamma and interleukin-2, tumor necrosis factor-alpha, and interleukin-6, initiating and maintaining the granulomatous response [23,24]. Interestingly, the memory CD4+ T cells in blood require a sustained CD28 costimulation for proliferative and cytokine responses to Be. However, intrapulmonary Be-specific CD4+ T cells do not express CD28, which ultimately would lead to a decreased proliferative capacity and an increased rate of apoptosis after stimulation with antigen. Using the ELISA method, only one team detected specific anti-Be antibodies in the serum of patients with CBD [25,26]. Figure 7 summarizes the pathogenesis of CBD.

Disease susceptibility is strongly linked with HLA-DPB1 alleles having a glutamic acid at position 69 of the β-chain (-Glu^69^) [27] or a βGlu^71^ expressing HLA-DR allele [28]. These polymorphisms would modify the conformation and electric charge of the antigen presentation pocket, enabling the binding of Be and disrupting the interaction with lymphocytes [27]. Carrying the HLA-DPβ1-Glu^69^ allele carries an increased CBD risk of 2–30-fold in Be-exposed workers. About 15% of CBD patients do not possess a Glu^69^-containing HLA-DP allele [29] but possess the HLA-DP-DRβ-Glu^71^ genotype [13].

### 3.4. Sensitization and Development of Berylliosis

Sensitization to Be (BeS) is a necessary condition but is not sufficient for the development of berylliosis [30]. The incidence of BeS, difficult to assess through systematic screening of exposed individuals, ranges from 2 to 19% [31,32]. The dose, composition, size of inhaled Be particles, duration of exposure, and individual factors determine the risk of BeS [18]. Extremely low environmental levels of Be exposure can induce sensitization and disease [33]. More than 50% of sensitized individuals would eventually develop berylliosis [34,35].

Berylliosis requires a latency period between exposure and the onset of the disease, which can range from 50 days [36] to 50 years with a median onset time of 10 years [37,38,39]. The onset of the disease is rarely sudden [40], but can be triggered by an infection, stress, pregnancy, or surgical intervention [41,42].

### 3.5. Clinical and Biological Features

Berylliosis is caused by a type IV delayed-type hypersensitivity to Be salts causing primarily a granulomatous disease of the lung. Berylliosis is a perfect phenocopy of sarcoidosis, both in terms of pulmonary involvement and extra-thoracic implications (see Table 2). The typical pathological findings of CBD are classically noncaseating granulomas and/or mononuclear cell infiltrates in the bronchial wall or lung interstitium. In some patients, the same pathology has been detected in the regional lymph nodes [43,44]. General symptoms are very common, sometimes prominent, including anorexia, night sweats, fever, and arthralgia [4,45]. Pulmonary involvement is said to be constant [4,46]. Cough, exertional dyspnea, and chest pain are said to systematically be accompanied by radiological abnormalities [40].

However, berylliosis is a systemic granulomatosis with possible involvement of extra-pulmonary organs [43]. Indeed, the skin, liver, myocardium, lymph nodes, spleen, skeletal muscles, bones, and salivary glands can be affected by the berylliosis granulomatous process [47,48]. Hepatic involvement is common, marked by the presence of granulomatous lesions and Be accumulation. It is often latent or accompanied by anicteric cholestasis or cytolysis [40,49]. Involvement of the atrioventricular conduction pathways and myocardium is rarer than in sarcoidosis [43]. Disordered vitamin D metabolism leading to hypercalcemia and nephrolithiasis or nephrocalcinosis is sometimes described [4,48]. Cases of chronic non-granulomatous interstitial nephritis have also been reported [50]. Rare cases of granulomatous involvement of the central nervous system [47], and nasal polyps have been described [51,52]. To our knowledge, no urothelial granulomatosis has ever been described.

The biological markers of berylliosis generally reflect immune system activation and redistribution of the lymphocyte pool to the affected organs. Therefore, it is common to observe polyclonal hypergammaglobulinemia as well as lymphopenia [53]. Additionally, hypercalciuria and/or hypercalcemia are sometimes present [54]. The angiotensin-converting enzyme is positive in 25% of cases of berylliosis, reflecting that macrophage activation and is correlated with disease severity [55].

**Table 2:** Summary of the main differences between sarcoidosis and CBD. Adapted from [56].

	Chronic Beryllium Disease	Sarcoidosis
Diagnosis	Exposition to beryllium. BeLPT (blood or bronchoalveolar lavage)	Exclusion diagnosis
Age and gender	Adults, young adults	Adults, young adults
Ethnicity	Not a risk factor	4–8 times more common in individuals of African descent.
Genetic susceptibility	HLA-DPB1 alleles with glutamic acid at position 69 of the β-chain (-Glu69), or βGlu71 expressing HLA-DR allele	HLA-DRB1*03, HLA-DRB1*07, DRB1*14 and DRB1*15
General symptoms	Commonly seen: Fever, night sweats, anorexia, unintentional weight loss, fatigue, arthralgia	30%: Fever, unintentional weight loss, fatigue, abdominal pain
Biological markers	BeLPT ACE positive in 25% of CBD (low specificity and sensitivity) Hypercalcemia, hypercalciuria	ACE (low specificity and sensitivity) Hypercalcemia, hypercalciuria normal to elevated 1,25-dihydroxyvitamin D, and normal to low 25-hydroxyvitamin D
Lung involvement	Constant. Frequent symptoms: Cough, exertional dyspnea, chest pain	More than 95%
Lymphadenopathy	Present (prevalence unknown)	Hilar or mediastinal lymphadenopathies are present in 90 to 98%
Kidney involvement	Rarely described. Tubulointerstitial nephritis (rarely granulomatous), nephrocalcinosis and lithiasis	2 to 10%: mainly nephrocalcinosis, renal stones. Granulomatous TIN in 20% (rarely alone)
Eye involvement	Not described in the literature	20–30% at presentation (25–90% of sarcoidosis): Uveitis represents 30–70% 40% of Granulomatous uveitis 15–38% of anterior bilateral uveitis

### 3.6. Diagnosis

Historically, the diagnosis of berylliosis was based on the presence of Be deposits in lung tissue showing granulomatosis [49]. However, it was later demonstrated that this presence only reflected exposure to the metal and not necessarily hypersensitivity to it [57]. Post mortem tissue assays of patients with CBD showed levels of Be in the lungs, but also detected Be in the hilar lymph nodes, liver, kidneys, spleen, myocardium, bones, and brain [41].

The development of the BeLPT, initially on blood samples and later on bronchoalveolar lavage (BAL), has transformed the diagnostic strategy and understanding of the disease [58,59]. The BeLPT is considered positive when lymphocytes are found to proliferate in a solution of Be sulfate [3]. Lymphocyte proliferation tests, like BeLPT, are based on the activation and expansion of specific memory T cells following their incubation with the suspected metal. The test was considered positive in our patient as stimulation index was >3 in the four highest tests from six tested Be concentrations. Several case series have demonstrated that the BeLPT, as an indicator of Be-specific cellular immune response, is abnormal in patients with berylliosis but not elevated in normal subjects, even those exposed to Be [60,61]. Aluminum sulfate is sometimes used as a negative control due to its implication in other pulmonary granulomatosis [18]. Skin testing is not recommended because of concern that such testing could either exacerbate disease or induce sensitization [60]. This test uses radiolabeled thymidine, which is integrated in the DNA during cell proliferation. In recent years, other in vitro markers have been used to diagnose delayed hypersensitivity reactions, in an effort to abandon this radioactive method. One of these is the flow cytometry measurement of a significant increase in activation marker CD69 on T cells after specific stimulation [62,63,64]. The sensitivity of BeLPT is 60 to 80% for blood samples [65] and 90 to 100% for samples from BAL [66]. These numbers are significantly reduced (38 to 71%) if the test is not conducted in a laboratory specialized in this field [67]. BeLPT may become negative after a decrease in beryllium exposure [68] and theoretically under corticosteroid treatment. There also seems to be false negatives in smokers. The intensity of lymphocyte proliferation is not correlated with the severity of the disease [60].

The diagnosis of berylliosis is confirmed by the combination of a positive BeLPT test and histologically proven granulomatosis [37,39].

### 3.7. Treatment and Outcome

Although no study has demonstrated its benefit, the cessation of Be exposure is recommended to limit its accumulation in the lungs [3]. No randomized data exist regarding the efficacy of treatments for berylliosis. Corticosteroids are the treatment of choice based on the similarity to sarcoidosis treatment. The classic corticosteroid initiation regimen follows that of sarcoidosis, typically 0.5 mg/kg/day of prednisone. However, higher doses are often required [41], for a longer duration, often lifelong [40], with relapses upon cessation [69]. Based on the experience provided with low-dose methotrexate therapy for sarcoidosis, this agent is often chosen for refractory CBD [70]. The mortality of berylliosis would range between 5 and 38% [71], due to terminal respiratory failure and chronic pulmonary heart [69]. Be is a recognized carcinogen, although data supporting its carcinogenicity in humans are derived from animal or in vitro data [72].

### 3.8. Summary

Our patient met the criteria for berylliosis, presenting with multi-organ granulomatous lesions and demonstrated lymphocytic sensitivity to beryllium. Chronic inhalation of Be salts in the workplace initiated the disease. His history of untreated sarcoidosis and multiple treatment for suspected tuberculosis without positive culture or PCR results are probably misdiagnosed manifestations of berylliosis. Surprisingly, his pulmonary symptoms were mild compared with his extra-thoracic organ involvement. Sustained urinary excretion and inflammatory response resulted in lesions mainly located in the urinary tract and kidneys, along with the lungs, but also possibly in the skin and sinuses, for which no biopsies were performed.

Retrospectively, renal mass biopsy prior to surgery would have been beneficial to avoid an ultimately unnecessary nephrectomy. Thirty percent of suspected renal masses would be benign and may not need an intervention. However, there is no global consensus on the management of T1a renal tumors [73].

Progressive renal dysfunction is likely multifactorial, having first experienced several episodes of obstructive acute kidney failure. Our patient also experienced episodes of tubulointerstitial nephritis, characterized by visible lymphocytic infiltration in the renal biopsy (Figure 3). Granulomas were not found in the renal sections examined, consistent with the literature, although they may have been present in other segments of the kidney that were not analyzed.

Finally, the present case illustrates the lack of robust epidemiological data about extra pulmonary berylliosis, mostly based on old case reports or small-scale autopsy results targeting the lungs. Renal and urinary tract granulomatous involvements have been probably missed because most berylliosis patients remain pauci-symptomatic.

## 4. Conclusions

CBD is a rare but underdiagnosed disease, often difficult to distinguish from sarcoidosis. Urothelial involvement in berylliosis has never been described to date. We describe a unique case of multisystemic berylliosis, dramatically associated with diffused urothelial granulomatous infiltration and pseudo-tumors, along with interstitial nephritis, ultimately responsible for end-stage kidney failure and the need for extra-renal epuration. This case illustrates the need to reconsider chronic berylliosis as a granulomatous disease less restricted to the lung than commonly believed. The progression of our patient to requiring dialysis could potentially have been mitigated with earlier identification and a broader consideration of the differential diagnosis, including conditions not traditionally associated with urological or nephrological presentations.

## Figures and Tables

**Table 1:** Laboratory findings.

Laboratory Findings	Patient’s Results	Normal Range
Hemoglobin (g/dL)	9.4	12–16
c-Reactive protein (mg/L)	15.3	<10
Serum creatinine (mg/dL)	5.7	0.7–1.2
Calcium (mmol/L)	2.41	2.20–2.55
Albumin (g/L) 25 (OH) Vitamin D (µg/L)	40 15.8	40–49 30–80
1-25 (OH)2-Vitamin D (pg/mL) Bioactive parathormone (ng/L)	38.3 560	29–84 <49
Serum angiotensin-converting enzyme (U/L)	54	8–55
Antinuclear antibody	Negative	Negative
ANCA ELISA anti MPO (U/mL) ELISA anti PR3 (U/mL) IgG 4 (mg/dL) C3 (g/L) C4 (g/L) C3d/C3 Hemolytic AP qualitative Classical pathway (CH50) (U/mL)	Negative <2 <2 22.8 1.33 0.29 0.95 Normal 88	Negative <20 <20 14–126 0.80–1.64 0.1–0.4 <1.4 Normal 41–94
Borrelia, bartonella, Hepatitis B, C, HIV, syphilis EBV, CMV, parvovirus B19	Negative	Negative
**Urinanalysis**	**Patient’s Values**	**Reference range**
Protein/creatinine ratio (g/g of creatinine)	0.57	<0.2
Albumin/creatinine ratio (mg/g of creatinine)	67.4	<30
Calcium (mmol/24 h)	4.0	2.5–7.5
Leucocytes (/µL)	129	<35
Erythrocytes (/µL) Glucose (mg/L) Casts/Crystals	<25 Absence Absence	<25 Absence Absence

## Data Availability

Data is contained within the article.
